# Increasing Extrinsic Motivation Improves Time-Based Prospective Memory in Adults with Autism: Relations with Executive Functioning and Mentalizing

**DOI:** 10.1007/s10803-019-04340-2

**Published:** 2019-12-21

**Authors:** Julia Landsiedel, David M. Williams

**Affiliations:** 1 Present Address: School of Psychology, Bangor University, Brigantia Building, Penrallt Road, Bangor, LL57 2AS UK; 2 School of Psychology, Keynes College, University of Kent, Canterbury, CT2 7NP UK

**Keywords:** Autism spectrum disorder, Prospective memory, Importance instructions, Executive functions, Mentalizing

## Abstract

Time-based prospective memory (PM) is diminished under various task demands in individuals with autism spectrum disorder (ASD). However, it is still unclear what underpins their impairment or how it could be remediated. This study explored whether instructions to prioritise one element of a PM task over another improved performance in adults with ASD (compared to a group of matched neurotypical adults), and how that is related to cognitive abilities. Results indicated that importance instructions significantly improved the PM performance of participants with ASD. Moreover, the extent of the benefit was associated significantly with objectively-measured executive set-shifting ability and self-reported inhibitory control ability (the poorer the set-shifting/inhibitory control, the *greater* the benefit). Implications for future research and clinical practice are discussed.

**Electronic supplementary material:**

The online version of this article (10.1007/s10803-019-04340-2) contains supplementary material, which is available to authorized users.

The ability to remember to carry out a delayed intention or plan at the appropriate moment in the future is known as prospective memory (PM; Kliegel et al. 2007). When PM requires to be carried out at a particular future time point, it is referred to as time-based (TBPM; Kliegel et al. 2008; Kvavilashvili and Ellis 1996). TBPM encompasses both remembering to carry out an action after a certain time delay (e.g., taking medication 30 min after eating), or at a specific time point (e.g., taking medication at 7 pm). As such, TBPM facilitates successful completion of daily tasks, although PM failures are common in the population (Terry 1988) and can have adverse consequences (Woods et al. 2015).

One neurodevelopmental disorder that is characterised by problems with TBPM is autism spectrum disorder (ASD). Previous research has found that both children and adults with ASD perform less well on TBPM tasks (for a review see Landsiedel et al. 2017). These difficulties arguably contribute to reduced everyday functioning and quality of life in ASD (van Heijst and Geurts 2015). However, to date, it is unclear why TBPM difficulties in ASD arise. This study explores the underlying causes of these difficulties by investigating ways to improve TBPM in ASD.

In laboratory experiments examining TBPM in neurotypical (NT) individuals, participants have to divide their attention between two tasks: an ongoing task (e.g., rating words on different dimensions) and a TBPM task, which usually requires participants to carry out a specific action at the appropriate moment (e.g., to press a response button every 2 min) (Einstein and McDaniel 1996; McDaniel and Einstein 2007). To successfully complete the TBPM task, participants need to monitor the elapsed time using a clock that is available on demand (e.g., by pressing another pre-specified keyboard key). A J-shaped function of time checks has been found to reflect strategic time monitoring processes (Mäntylä and Carelli 2006), with frequent initial time checks, followed by a period of few time checks, and then a linear increase in checks as the target time approaches. Thus, TBPM involves a series of self-initiated processes to enable PM retrieval (Einstein et al. 1995). It is thought that several elements of executive functioning (EF) work together to support this process. Working memory supports the maintenance of the time-based intention while participants complete the ongoing task and track the passage of time (Mioni and Stablum 2014; Voigt et al. 2014). Inhibitory control, as well as cognitive flexibility, are required to interrupt attention to the ongoing task (Kerns 2000) and either initiate a time-check (Mioni and Stablum 2014; Mioni et al. 2012; Vanneste et al. 2016) or to execute the PM intention (Kliegel et al. 2002; Kliegel et al. 2003; Mackinlay et al. 2009). Finally, mentalizing/theory of mind (the ability to represent one’s own and others’ mental states; Frith and Frith 2005) has also been implicated in TBPM, because it requires the ability to recognise one’s own *intention* to act (e.g., Williams et al. 2013); if one has difficulty recognising and reflecting on one’s own intentions, then it may be difficult to carry out one’s pre-formed PM intention.

One critical aspect encoded during PM intention formation is the importance level attached to completing the intention (Kvavilashvili and Ellis 1996; Walter and Meier 2014). Thus, an intention’s importance will be a crucial determinant of successful PM performance in everyday life, as well as in experimental tasks. For example, collecting lifesaving medication for one’s child is likely to be undertaken regardless of the costs to one’s ongoing activities (e.g., even if it makes one late for work), whereas collecting one’s dry-cleaned clothes may not be important enough to disrupt one’s ongoing daily activities for. In everyday life, the importance of completing an intended action will vary depending on personal (e.g., desires, goals, other intentions) and social (e.g., self- or other-generated intentions) factors. In lab-based experiments, however, perceived importance is based on how a particular task is instructed. Typically, participants receive a standard instruction; for example, “please remember to press this button in 2-min intervals”. However, no indication is usually given by the experimenter about how important the PM intention is *relative to* the importance of the ongoing task. In this context, participants need to infer the experimenter’s implicit expectation about how to perform a TBPM task alongside the ongoing task. Specifically, participants have to decide whether this instruction means that both ongoing task and TBPM task are equally important or whether either of the two is more important relative to the other. Thus, mentalizing also contributes to successful TBPM with regards to understanding the demands of *others* when they give TBPM instructions.

Crucially, *relative importance* of an intention (i.e., the importance of the intention to perform an action relative to the intention to perform a second action) affects PM performance by increasing extrinsic motivation (doing something for external incentives/driving factors). This leads to changes in the strategic allocation of attentional resources towards the completion of the intended action, even if it is at the expense of other ongoing activities (Walter and Meier 2014). For TBPM, only one study has so far explored the effect of relative importance instructions on task performance (Kliegel et al. 2001). For the ongoing task, participants rated single words on one of four aspects (concreteness, familiarity, pleasantness, and seriousness). For the TBPM task, participants had to remember to press a pre-specified key in 2-min intervals. The task lasted just under 9 min and involved four PM target periods (at minutes 2, 4, 6 and 8). A PM hit was given if participants responded within ± 2.5 s of the respective target time. Half of the participants were told that the TBPM task was more important than the ongoing task (PM high importance group), while the other half were told that the ongoing task was more important than the TBPM task (PM low importance group). Results revealed that emphasising TBPM had a large positive effect on PM hit rates (*d *= 1.14) due to the increased timeliness of the PM responses; i.e. more PM responses within the target interval (*d *= 1.11). Time monitoring was also increased in the time window close to the PM target time indicating a strategic allocation of attentional resources leading to higher PM accuracy. Interestingly, the costs of high PM importance on the ongoing task, defined as not giving a rating for a word on a given trial, were restricted to trials prior and after a PM target time, instead of having an overall effect on word rating performance. Other studies into the effects of relative importance have focused on a different type of PM (i.e.; event-based PM; e.g.; Loft et al. 2008; Smith and Bayen 2004). This research further supports beneficial effects of emphasising the PM task over the ongoing task in typical development (see S1 for more details on these studies, or Walter and Meier 2014 for a comprehensive review).

Clear task instructions with regards to the importance of a delayed intention may be particularly relevant in ASD given their EF problems (Hill 2004; Kenworthy et al. 2008). For instance, individuals with ASD might struggle to inhibit attending to the ongoing task and to shift attention to the passage of time. A change in strategic allocation of attention might compensate these problems and lead to a performance benefit for individuals with ASD. However, only inconsistent or no associations have been found between TBPM and measures of EF that are clearly associated with TBPM in NT individuals. Henry et al. (2014) reported associations of TBPM with a combined measure of inhibition and task switching whereas other studies found no association with cognitive flexibility or working memory in ASD (Williams et al. 2013; Williams et al. 2014). Hence, the underlying neurocognitive basis of TBPM difficulties in ASD remains unclear.

One explanation for the unclear role of specific EF for TBPM in ASD might be that it is not EF problems per se that cause poor task performance. White (2013) proposes that difficulties among people with ASD on many cognitive tasks (particularly tests of executive control) result from a limited ability to infer the experimenter’s expectation for what is required on the measure (Triple I hypothesis). Therefore, tasks that are unstructured, open-ended, or that require the inference of the experimenter’s implicit expectations will result in difficulties for individuals with ASD. White (2013) made this case specifically to explain the variability of performance on EF tasks in ASD. However, the Triple I hypothesis equally applies to TBPM tasks where participants are not explicitly told about the importance of the PM component. Mentalizing problems in ASD might result in difficulties to form a clear representation of the experimenter’s implicit expectation that carrying out the TBPM intention is a crucial part of completing the task, and thus result in TBPM impairments in ASD. Williams et al. (2013) provided tentative evidence for this idea.

Therefore, the main aim of the current study was to investigate whether manipulation of relative importance of delayed intentions would influence TBPM task performance in ASD. Participants with and without a diagnosis of ASD completed two versions of a TBPM task in counterbalanced order. One version emphasised the importance of the PM task over the ongoing task (PM high importance condition) and the other version emphasised the importance of the ongoing task over the PM task (PM low importance condition).

Depending on the mechanism of our main task manipulation, two different predictions were made: (1) If problems with the executive demands of TBPM underlie poorer PM performance in ASD, a Group (ASD/NT) × Condition (PM high importance/PM low importance) interaction effect on TBPM performance would occur. Specifically, individuals with ASD would show increased performance from the PM low to PM high importance condition compared to NT participants. (2) If problems with mentalizing underlie poor PM performance in ASD, benefits of task instruction would not be significantly greater in the ASD compared to the NT group.

We also furthermore explore potential cognitive correlates related to *changes in* TBPM performance across conditions. Behavioural measures of inhibition and cognitive flexibility as well as mentalizing abilities were assessed. It was predicted that ASD participants with lower performance on these tasks would benefit more from the structure-enhancing importance instruction. Additional self-report measures were used to explore whether individuals who report poorer EF and PM in everyday life are those who will benefit most from the importance manipulation or whether the manipulation has (if any) effects regardless of how individuals perceive their own abilities.

## Methods

### Participants

Twenty-five participants with ASD (21 male) and 23 NT control participants (19 male) were recruited for this study. The study was approved by the ethics committee of the School of Psychology at the University of Kent and participants gave written informed consent before taking part. All participants in the ASD group had a confirmed diagnosis of ASD according to standard diagnostic criteria (ICD-10, DSM-IV or DSM-5) (American Psychiatric Association 2000, 2013; World Health Organization 2006). Additionally, all participants with ASD undertook the Autism Diagnostic Observation Schedule, second edition (ADOS-2, Lord et al. 2012), scores on which provide an indication of ASD severity. All participants completed the Autism-spectrum Quotient (AQ), a self-report measure of ASD traits (Baron-Cohen et al. 2001). The established screening cut-off for ASD is a total score of 26 (Woodbury-Smith et al. 2005). Groups were matched closely for chronological age and gender, as well as for verbal IQ, performance IQ, and full-scale IQ using the Wechsler Abbreviated Scale of Intelligence (WASI-II, second edition, Wechsler 2011; see Table 1).

**Table 1:** Sample characteristics

	Group means (SD)		*T*	*df*	*p*	Cohen’s *d*
	ASD (*n* = 25)	NT (*n* = 23)				
Age	34.84 (11.42)	38.24 (13.19)	− 0.96	46	0.34	− 0.28
VIQ	105.20 (13.64)	104.35 (9.60)	0.25	46	0.81	0.07
PIQ	102.92 (19.39)	104.65 (10.81)	− 0.38	46	0.71	− 0.11
FSIQ	104.32 (16.44)	104.87 (9.39)	− 0.14	46	0.89	− 0.04
AQ	31.00 (9.06)	16.91 (6.75)	6.07	46	< .001	1.76
ADOS	8.80 (4.09)					

### Materials and Procedures

#### Prospective Memory Task

Participants completed two conditions of a computerised TBPM experiment in counter-balanced order to explore effects of importance instructions. The same ongoing and PM task was used in each condition. Each condition lasted for approximately 11 min and contained 40 ongoing trials and five PM trials. The conditions differed only in how their importance was instructed. In the *PM low importance condition*, participants were told that the ongoing task was more important than the PM task, whereas in the *PM high importance condition* instructions were reversed, i.e., the PM task was instructed to be more important than the ongoing task. Between the two conditions, there was a 45-min gap during which participants completed several filler tasks.

**Fig. 1:**
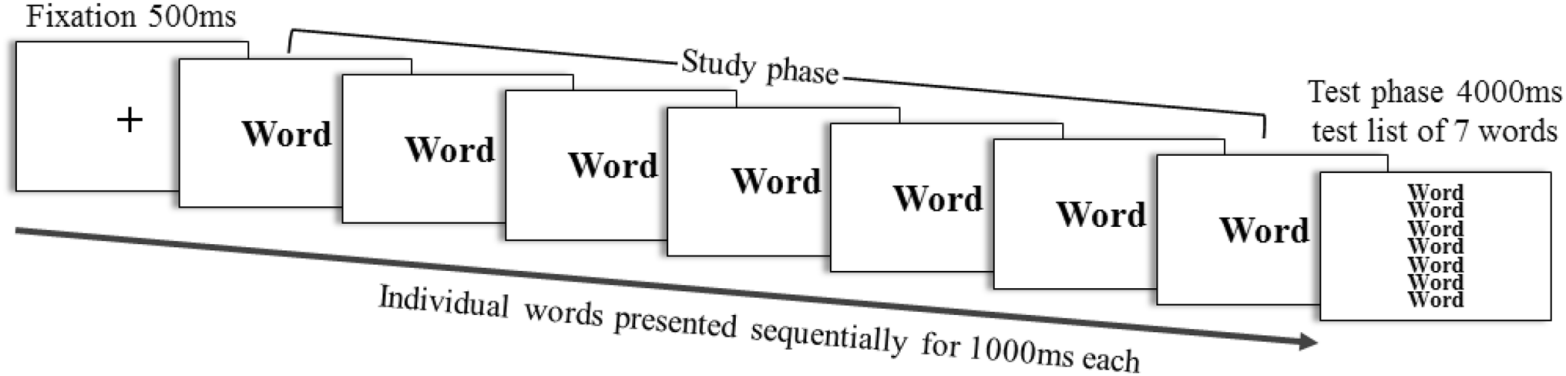
Schematic depiction of the trial structure of the ongoing task

The *ongoing task* (see Fig. 1) followed the procedure previously used by Williams et al. (2014). Each trial consisted of a study phase and a test phase. In the study phase, participants had to memorise seven words which were presented sequentially in the centre of a computer screen. In the test phase, a list of seven words appeared for 4 s. Participants had to indicate whether all the words in the test list matched the seven words presented during the study phase, and to press the respective keyboard key in response (yes/no answer). In half of the trials, the words in the test phase were identical to those during the study phase and for the other half of the trials, one word was replaced with a lure item that had to be found. To ensure understanding of task instructions, participants had to explain the task to the experimenter in their own words. Furthermore, participants had to complete five practice trials. The experimenter provided verbal feedback to the participant once the practice trials were completed. If a participant still expressed doubts about how to perform the ongoing task, the experimenter explained again and there was another chance to perform an additional five practice trials.

A score of one was awarded for each correct response and a proportion score (*ongoing task proportion hits*) was calculated, as the total number of hits relative to the total number of ongoing trials. For the analysis of the performance change between the two conditions, a difference score was computed by subtracting performance in the PM high importance condition from the performance in the PM low importance condition. A positive difference score indicates better ongoing performance in the PM low importance condition.

Two sets of 300 words were used as stimuli counter-balanced across conditions (280 word stimuli plus 20 lure stimuli). Both sets were equated for frequency and syllable length according to Kucera and Francis (1967) guidelines and derived from the MRC Psycholinguistic Database (Coltheart 1981). The matching of the sets was confirmed using multivariate analyses finding a nonsignificant main effect of set, Wilk’s criterion, *F*(2,557) = 0.14, *p *= .87.

For the embedded *PM task*, participants were instructed to press a pre-specified keyboard key in 2-min intervals (PM target times at minutes 2, 4, 6, 8, and 10). To keep track of the time, a digital clock could be displayed on the screen (lasting for 1.5 s before disappearing again) by pressing a pre-specified keyboard key.

Retrospective memory for task instructions was checked after each condition, specifically participants had to correctly recall which of the two tasks the more important one was. All participants successfully retrieved the PM instruction.

The scoring of task performance followed that of Williams et al. (2014). A score of one was awarded for each PM hit. A PM hit was defined as pressing the pre-specified key within a 15 s time window around each target time (± 7.5 s). This scoring criterion ensured that a participant would have had enough time to respond to both the ongoing and PM task on any trial even if the PM target time fell in the middle of a trial (see Williams et al. 2014). A proportion score (*PM proportion hit*) *was* calculated as the number of PM hits out of five PM trials. Additionally, *PM target accuracy* was calculated as the mean of the absolute difference between the time of remembering the PM task (i.e., pressing the pre-specified key) and the target time across all PM hit trials. For example, a PM hit at 2 min and 5 s (125 s) would result in a negative PM target accuracy for that trial of minus 5 s. When averaging across all PM hit trials, the absolute value is used; e.g., |120–125 s| = 5 s for each trial because otherwise one would distort the mean by averaging across positive and negative values. A value of zero would indicate perfect accuracy (i.e., pressing exactly on the target time), and hence the bigger the value the further from target time the PM press happened. For the analysis of time-monitoring behaviour, the five 2-min periods prior to each target time were each broken down into 30-s segments and the mean number of time checks in each time interval (0–30, 31–60, 61–90, 91–120 s) across all five PM trials was calculated.

To assess correlates of change in TBPM performance from one condition to the other, *difference scores* were computed for each of the dependent variables (proportion hit scores, target accuracy, and mean number of time checks in the critical fourth interval prior to target time) by subtracting performance in the PM low importance condition from performance in the PM high importance condition. Positive difference scores for proportion accuracy and mean number of time checks indicate increased performance in the high importance condition (i.e., higher number of hits/more time checks in the PM high condition). Negative difference scores for target accuracy indicate that participants responded closer to target time in the PM high importance condition.

#### Self-report Measures

Behaviour Rating Inventory of Executive Function—Adult version (BRIEF-A; Roth et al. 2005): The BRIEF-A was administered to assess self-report everyday EFs. The BRIEF-A consists of 75 statements describing specific behaviours and participants have to rate how often a certain behaviour has been a problem within the past month on a 3-point scale (‘Never’, ‘Sometimes’, ‘Often’). Several subscales (Inhibit, Shift, Emotional control, Self-monitor, Initiate, Working memory, Plan/organise, Task monitor, and Organisation of materials) and summary scores (Behaviour regulation index, Metacognition index, and Global composite score) can be calculated. Of particular interest for this study are the Inhibit subscale (measuring the ability to control prepotent responses/inhibitory control) and Shift subscale (measuring the ability to switch flexibly between activities or situations, to switch attentional focus; and to tolerate change). Raw scores were converted to age-normed T-scores (with a mean of 50 and a SD of 10) with higher scores indicating greater executive problems. Scores above a T-score of 65 are considered as potentially clinically relevant.

Prospective Retrospective Memory Questionnaire (PRMQ, Crawford et al. 2003; Smith et al. 2000): The PRMQ is a self-report measure of everyday memory slips. It is composed of 16 items probing for both retrospective and prospective memory difficulties. Participants have to rate the frequency of described memory slips on a 5-point scale (‘Very often’, ‘Quite often’, ‘Sometimes’, ‘Rarely’, ‘Never’). Sum scores provide a general memory factor, as well as a prospective and retrospective memory score. Raw scores were converted to standardised T-scores (Crawford et al., 2003), with higher T-scores indicating better memory.

#### Behavioural Measures

A computerised version of the colour-word *Stroop task* (Stroop 1935) was used to measure inhibitory control (Miyake et al. 2000). Participants had to indicate the correct colour of a word as quickly as possible irrespective of whether its meaning matched (congruent trials), mismatched (incongruent trials) or was unrelated to (neutral trials) the word colour. Inhibitory control was operationalised as the reaction time difference between correct responses to incongruent versus congruent trials.

A computerised version of the *Wisconsin Card Sorting Task* (WCST; Berg 1948; Nelson 1976) was used to measure cognitive flexibility/set shifting (Miyake et al. 2000). Participants had to sort cards that varied along three dimensions into categories according to an unknown rule using trial-by-trial feedback. The sorting rule changed unbeknown to the participant after 10 correct sorts. The number of perseverative errors was used as an index of cognitive inflexibility (i.e., a tendency to become stuck in set). A perseverative error was defined as persisting to sort a card into the same category as the previous one, after they had received feedback of their previous sort being incorrect (see Cianchetti et al. 2007).

The *Animations task* (Abell et al. 2000) was used to measure mentalizing. Participants had to describe four short video clips. Each clip showed the interaction between two triangles. An accurate description of the scene required the attribution of mental states to the triangles. The accuracy of the descriptions was coded following the guidelines by Abell et al. (2000) with scores ranging from zero to two for each clip. Hence, the mentalizing total score ranged from zero to eight.

Further details of the behavioural tasks can be found in Supplementary Materials S2.

### Statistical Analysis

An alpha level of .05 was used to determine statistical significance. Where ANOVAs were used, partial eta squared ($\eta_{p}^{2}$) is reported as a measure of effect size (≥ .01 = small effect, ≥ .06 = moderate effect, ≥ . 14 = large effect; Cohen 1988). Where *t*-tests were used, Cohen’s *d* is reported as a measure of effect size (≥ .0.20 = small effect, ≥ 0.50 = moderate effect; ≥ 0.80 = large effect; Cohen 1988).

### Bayes Factor Analysis

An increasingly used alternative to power calculations is to calculate a Bayes factor associated with the critical result of interest. According to standard statistical conventions, if a *p* value for a specific analysis is > .05 then it can be concluded that the result is null and does not support the alternative hypothesis. However, treating *p* values in this way assumes they are categorical and absolute, rather than “a convenient reference point along the possibility-probability continuum’’ (Cohen 1990, p. 1311). Bayes factors overcome this issue by estimating the relative strength of a finding for one theory over another theory, allowing a more graded interpretation of the data (e.g., Rouder et al. 2009). Thus, they are especially useful for interpreting null results, because they provide an estimate of the degree to which findings are supportive of the null hypothesis (H_0_) *over* the alternative hypothesis (H_1_) (Dienes 2014). Therefore, additionally to the traditional null hypothesis significance testing approach, Bayes factors were calculated. According to Lee and Wagenmakers (2013) adjusted criteria of Jeffreys’ (1961) original criteria for interpreting Bayes factors, values larger than 10 provide strong evidence for H_1_, values between three and 10 provide moderate evidence for H_1_, and values between one and three provide anecdotal evidence for H_1_. A Bayes factor of one does not provide any evidence in favour of either the H_1_, or H_0_. Values between one-third and one provide anecdotal, between one-tenth and one-third moderate, and smaller than one-tenth strong evidence for H_0_. Thus, Bayes factors between one-third and three provide inconsistent evidence for the respective hypothesis.

Bayesian analyses were computed using the software JASP (Version 0.10.0.0) using the recommended default objective prior for the respective analysis (JASP Team, 2017; Wagenmakers et al. 2017a; b).

## Results

### Sample Description

Table 2 shows the groups’ performance/scores for the self-report and behavioural measures of mentalizing and EF. Due to technical problems during data collection, only 21 participants in each group completed the WCST, and 21 ASD participants as well as 20 NT participants completed the Stroop task. Furthermore, only 22 NT participants completed the BRIEF-A. We ensured that participants in the experimental groups remained matched for baseline characteristics before conducting the respective correlational analyses in these sub-samples. On both self-report measures (PRMQ and BRIEF-A), the ASD group reported significantly more problems relative to the NT group, with large associated effect sizes. Lower scores on the PRMQ subscales indicate both prospective and retrospective memory problems in the ASD group. Higher scores on the two BRIEF scales indicate more executive problems in everyday life in the ASD group. Furthermore, the ASD group showed poorer cognitive flexibility on the WCST, as indexed by a greater number of perseverative errors. Finally, the ASD and comparison groups performed equally well on the Stroop and Animation tasks.

**Table 2:** Sample characteristics for behavioural and self-report measures of mentalizing, PM, and executive functions

	Group means (SD)		*T*	*df*	*p*	Cohen’s *d*
	ASD	NT				
Mentalizing (animations task)	4.64 (1.82)	5.30 (1.82)	− 1.26	46	.21	− 0.36
PRMQ prospective memory scale	40.60 (12.43)	50.96 (10.39)	− 3.12	46	.003	− 0.90
PRMQ retrospective memory scale	45.56 (9.16)	53.61 (8.85)	− 3.09	46	.003	− 0.89
BRIEF inhibit	63.40 (12.47)	51.82 (9.81)	3.50	45	.001	1.02
BRIEF shift	72.04 (13.82)	50.59 (8.16)	6.37	45	<.001	1.86
WCST perseverative errors	13.52 (10.98)	5.62 (4.40)	3.06	40	.004	0.95
Stroop inhibitory control	105.71 (88.14)	84.66 (134.47)	0.60	39	.56	0.19

There was some missing data for executive function measures. Only 22 NT participants completed the BRIEF-A, 21 participants in each group completed the WCST, and finally, 21 ASD participants and 20 NT participants completed the Stroop task. Participants in the experimental groups remained matched for baseline characteristics for all correlational analyses. Sample characteristics for the remaining scales of the BRIEF-A can be found in Supplementary Materials S3

### Ongoing Task

Table 3 shows the mean (SD) for ongoing task performance among each diagnostic group. This data was subjected to a 2 × 2 mixed ANOVA with Group (ASD/NT) as between-subject factor and Condition (PM low/high importance) as within-subject factor. There were significant main effects of Group, *F*(1,46) = 12.63, *p* = .001, $\eta_{p}^{2}$ = .22, BF_10_ = 33.08, and Condition, *F*(1,46) = 5.30, *p* = .03, $\eta_{p}^{2}$ = .10, BF_10_ = 2.00. The NT group performed better overall than the ASD group, and both groups showed better ongoing performance in the PM low importance condition than in the PM high importance condition. No significant interaction effect emerged, *F*(1,46) = 0.17, *p* = .68, $\eta_{p}^{2}$ = .004, BF_10_ = 0.28. The BF_10_ for the additive model was 67.51 and for the full model 18.96. This means that the additive model provided the greatest evidence for the present data. Group differences on the ongoing task indicate that the ASD group might have had fewer attentional resources to perform the PM task compared to the NT group. This could be problematic for the interpretation of any group differences in PM performance. Therefore, all analyses were repeated among a subsample that was matched for ongoing task performance as well as baseline characteristics (see Supplementary Materials S4). Crucially, the results concerning PM performance were not substantially different in this subsample (matched for ongoing task performance) and in the full sample (unmatched for ongoing task performance). Therefore, the results from the full sample are reported here. The full details of the subsample analyses can be found in the Supplementary Materials (S4).

**Table 3:** Ongoing and PM performance scores by group and condition

Condition	Group means (SD)	
	ASD (*n *= 25)	NT (*n *= 23)
PM low importance		
Ongoing task proportion hits	.65 (0.15)	.78 (0.10)
PM proportion hits	.69 (0.34)	.95 (0.09)
PM target accuracy*	2.24 (1.60)	1.37 (1.05)
Time checks 4th interval	1.70 (0.92)	2.17 (1.27)
PM high importance		
Ongoing task proportion hits	.63 (0.12)	.74 (0.13)
PM proportion hits	.91 (0.21)	.99 (0.04)
PM target accuracy*	1.66 (1.00)	1.02 (0.85)
Time checks 4th interval	2.26 (1.02)	3.21 (1.67)

*n = 23 in each group

### PM Task

#### PM Hits

**Fig. 2:**
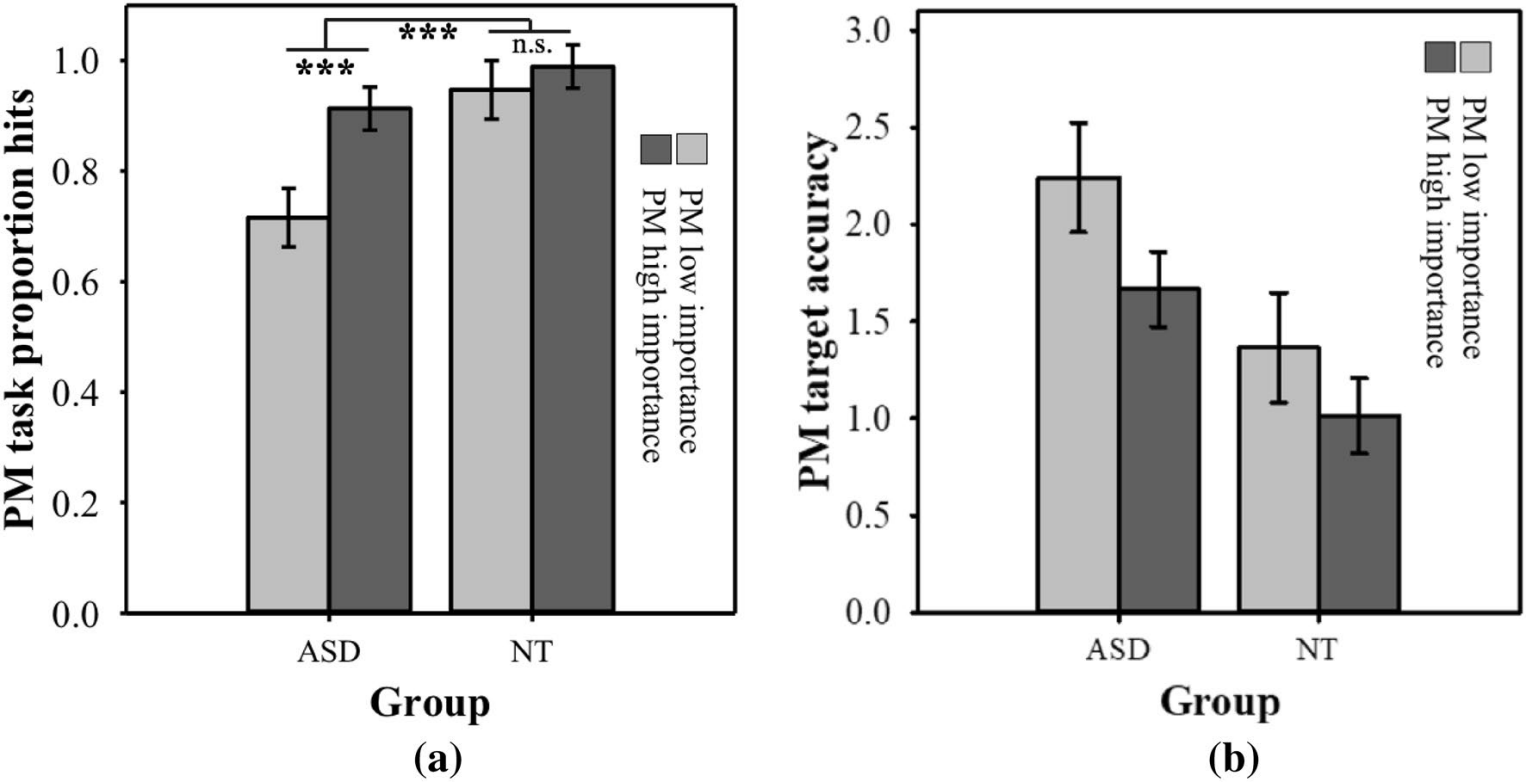
PM performance by group and condition for: **a** PM proportion hits; **b** PM target accuracy; Error bars represent standard errors of the means. ***p ≤ .001, *n.s.* not significant

Table 3 shows the mean (SD) for PM accuracy for each diagnostic group. PM proportion hit scores were used as the dependent variable in a 2 × 2 mixed ANOVA with Group (ASD/NT) as between-subjects factor and Condition (PM low/high importance) as within-subject factor. There were significant main effects of Group, *F*(1,46) = 10.95, *p* = .002, $\eta_{p}^{2}$ = .19, BF_10_ = 16.82, and Condition, *F*(1,46) = 18.09, *p* < .001, $\eta_{p}^{2}$ = .28, BF_10_ = 141.50. More importantly, however, there was a significant interaction between Group and Condition, *F*(1,46) = 8.18, *p* = .006, $\eta_{p}^{2}$ = .15, BF_10_ = 6.63 (see Fig. 2a). The BF_10_ for the additive model was 2639.19 and for the full model 17503.47, indicating that the full model provided the greatest evidence for the present data. Tests of simple effects comparing PM performance within groups revealed that the proportion of PM hits was significantly higher in the PM high importance condition than in the PM low importance condition among participants with ASD, *F*(1,46) = 26.40, *p* < .001, $\eta_{p}^{2}$ = .37, *d* = 0.86; BF_10_ = 52.74, but not among NT participants: *F*(1,46) = 0.93, *p* = .34, $\eta_{p}^{2}$ = .02, *d* = 0.44, BF_10_ = 1.20. Tests of simple effects of between-group differences indicated that ASD participants performed significantly less well than the comparison group in the PM low importance condition, *F*(1,46) = 12.81, *p* = .001 $\eta_{p}^{2}$ = .22, *d* = − 1.05, BF_10_ = 36.53. In the PM high importance condition, the ASD group again performed less well that the NT group, although the difference did not reach statistical significance and was associated with a Bayes factor that provided little evidence in favour of the alternative hypothesis, *F*(1,46) = 3.32, *p* = .08, $\eta_{p}^{2}$ = .07, *d* = − 0.53, BF_10_ = 1.09. However, post hoc analysis showed that the proportion of PM hits among the NT group was non-significantly below ceiling in the PM high importance condition, *t*(22) = − 1.00, *p* = .33, d = − 0.21, BF_10_ = 0.34. Thus, while the finding that PM performance among participants with ASD improved significantly across conditions is valid and important, caution should be taken when drawing the conclusion that the ASD group improved significantly *more* across conditions than did the NT group.

#### PM Target Accuracy

Table 3 shows the mean (SD) for PM target accuracy for each diagnostic group. Two participants in the ASD group were not included in this analysis as they didn’t have at least one PM hit in each condition (ASD *n *= 23, NT *n* = 23). PM target accuracy was analysed in a 2 × 2 mixed ANOVA, with Group (ASD/NT) as between-subjects factor and Condition (PM low/high importance) as within-subject factor. A main effect of Group emerged indicating that the NT group overall responded closer to target time than the ASD group, *F*(1,44) = 6.94, *p* = .01, $\eta_{p}^{2}$ = .14, BF_10_ = 4.20. Furthermore, a significant main effect of Condition indicated that both groups responded closer to target time in the PM high importance condition than in the PM low importance condition, *F*(1,44) = 6.36, *p* = .02, $\eta_{p}^{2}$ = .13, BF_10_ = 3.21. However, the Group × Condition interaction did not approach significance, *F*(1,44) = 0.38 *p* = .54, $\eta_{p}^{2}$ = .01, BF_10_ = 0.32 (see Fig. 2b). The BF_10_ for the additive model was 14.43 and for the full model 4.59. This means that the additive model provided the greatest evidence for the present data.

#### Time-Monitoring

**Fig. 3:**
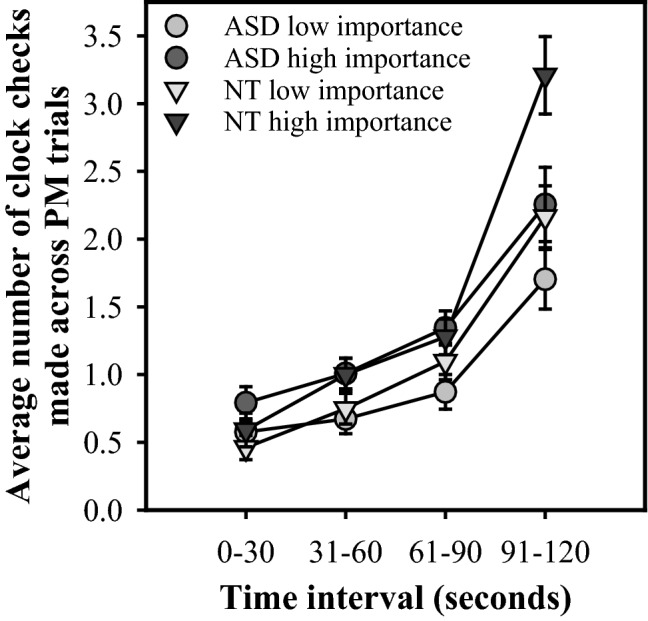
Time-monitoring pattern by time interval in the ASD and NT group by importance condition

Figure 3 shows the mean number of time checks carried out for each condition during each of the four time periods among ASD and comparison participants. A 2 × 2 × 4 mixed ANOVA was conducted on this data with Group (ASD/NT) as between-subjects factor, and Condition (PM low/high importance) and Time interval (0–30, 31–60, 61–90, 91–120 s) as within-subject factors. The main effect of Group was non-significant, *F*(1,46) = 1.08, *p* = .30, $\eta_{p}^{2}$ = .02, BF_10_ = 0.29. There were significant main effects of Condition, *F*(1,46) = 23.01, *p* < .001, $\eta_{p}^{2}$ = .33, BF_10_ = 348.61, and Interval *F*(1.39,63.75) = 122.23, *p* < .001, $\eta_{p}^{2}$ = .73, BF_10_ = 2.02 × 10^49^. The Group × Importance interaction was non-significant, *F*(1,46) = .002, *p* = .96, $\eta_{p}^{2}$ < .001, BF_10_ = 0.17, but there were significant two-way interactions between Group and Interval, *F*(1.39,63.75) = 7.36, *p* < .004, $\eta_{p}^{2}$ = .14, BF_10_ = 1230.52, and Condition and Interval, *F*(2.11,97.19) = 9.09, *p* < .001, $\eta_{p}^{2}$ = .17, BF_10_ = 4.84. Most importantly, all these effects were qualified by a significant three-way Group × Importance × Interval interaction *F*(2.11,97.19) = 3.41, *p* = .04, $\eta_{p}^{2}$ = .07, BF_10_ = 0.35. Of all the different models possible, the one with all three main effects and the two significant two-way interactions described above attained the largest Bayes factor,^1^ BF_10_ = 1.56 × 10^59^. Tests of simple effects revealed a significant between-group difference in number of time checks in the crucial interval immediately prior to target time (91–120 s) in the PM high importance condition only; in this condition, significantly more time checks were made in the NT group (*M* = 3.21, *SE* = 29) than in the ASD group (*M* = 2.26, *SE* = .27), *F*(1,46) = 5.79, *p *= .02, $\eta_{p}^{2}$ = .11, BF_10_ = 2.83. Furthermore, when comparing time checks for each group, the ASD group checked the time significantly more often for three intervals (31–60, 61–90, 91–120 s) in the PM high importance condition (all *F*s ≥ 5.39, all *p*s ≤ .03, all $\eta_{p}^{2}$ ≥ .11, all BF_10_ ≥ 5.05) compared to the PM low importance condition, whereas the NT group only checked the time more often in intervals 31–60 and 91–120 (all *F*s ≥ 4.48, all *p*s ≤ .04, all $\eta_{p}^{2}$ ≥ .09, interval 31–60 BF_10_ = 1.15, interval 91–120 BF_10_ = 24.39) in the PM high vs PM low importance condition.

### Cognitive Correlates

#### Ongoing Task

Ongoing task performance was collapsed across conditions for each group before being entered into correlation analyses. Additionally, to explore correlates of change in ongoing task hits between conditions, a difference score was computed (positive values indicating better ongoing performance in the PM low importance condition). No relations between EF measures (indexed by self-reported EF problems on the BRIEF-A, as well as performance on the WCST and Stroop) and ongoing task performance or the change in performance were found in either group. The only significant correlation was found between mentalizing and ongoing task performance in the ASD group (see Table 4). Therefore, ongoing task performance was partialled out when investigating the correlation between mentalizing and change in PM performance.

**Table 4:** Correlates of change (Δ) in PM and ongoing performance, and ongoing performance overall

	Group		Δ PM proportion hit	Δ Target accuracy	Ongoing task proportion hits (collapsed)	Δ Ongoing task proportion hits
Δ I4 time monitoring	*ASD*	*r*	.40*	− .45*	.05	− .12
		BF_10_	1.62	2.21	0.57	0.39
	*NT*	*r*	− 0.27	− .34	.23	.32
		BF_10_	0.53	0.86	0.27	1.43
BRIEF inhibition scale	*ASD*	*r*	.23	− .66***	− .15	.02
		BF_10_	0.45	58.02	0.32	0.25
	*NT*	*r*	− .16	− .12	− .24	− .23
		BF_10_	0.33	0.30	0.46	0.44
BRIEF shifting scale	*ASD*	*r*	.01	− .24	− .12	− .11
		BF_10_	0.25	0.46	0.29	0.28
	*NT*	*r*	− .25	.11	.07	− .31
		BF_10_	0.49	0.29	0.28	0.67
WCST perseverative errors	*ASD*	*r*	.61**	− .19	− .25	.02
		BF_10_	14.93	0.37	0.46	0.27
	*NT*	*r*	.28	− .15	.22	.26
		BF_10_	0.54	0.33	0.42	0.50
Stroop inhibition index	*ASD*	*r*	<.01	− .26	< .01	− .17
		BF_10_	0.27	0.49	0.27	0.35
	*NT*	*r*	− .06	.04	− .20	− .30
		BF_10_	0.28	0.28	0.38	0.58
Mentalizing score (animations task)	*ASD*	*r*	− .27	− .15	.48*	− .15
		BF_10_	0.55	0.33	3.95	0.31
	*NT*	*r*	.22	.35	.28	.33
		BF_10_	0.41	0.95	0.58	0.78
PRMQ prospective memory scale	*ASD*	*r*	− .10	.39^†^	− .21	.18
		BF_10_	0.28	1.32	0.40	0.35
	*NT*	*r*	.08	− .12	− .03	− .07
		BF_10_	0.27	0.27	0.26	0.27
PRMQ retrospective memory scale	*ASD*	*r*	− .05	.46*	− .09	.19
		BF_10_	0.26	2.68	0.27	0.36
	*NT*	*r*	.09	− .37	.02	− .08
		BF_10_	0.29	0.28	0.26	0.27

Correlations significant two-tailed ^†^p ≤ .06, *p ≤ .05, ***p ≤ .001

#### PM Task

Difference scores were computed for proportion hit scores, target accuracy, and time checks in the critical last interval. Subsequently, a series of correlations was performed between indices of TBPM performance change across conditions and (a) time monitoring; (b) self-report and behavioural measures of EFs; (c) a self-report measure of memory; and (d) a behavioural measure of mentalizing. Furthermore, correlations explored whether modulation of time monitoring frequency was associated with any of these measures (see Table 4 for all the results of the correlation analyses and the associated Bayes factors).

The partial correlation between change in PM proportion hits across conditions and perseverative errors on the WCST controlling for non-perseverative errors was significant in the ASD (*r* = .47, *p *= .04, BF_10_ = 2.36) but not in the NT group (*r* = .25, *p *= .29, BF_10_ = 0.47). However, a Fisher *z*-test revealed that the size of the correlation in the ASD group was not significantly different from the one in the NT group (*z* = 0.76, *p* = .45). The partial correlation between change in PM proportion hits and mentalizing (Animations task theory of mind score) controlling for overall ongoing task performance was not significant in either group (ASD: *r* = **-**.33, *p *= .11, BF_10_ = 0.88; NT: *r* = .16, *p *= .47, BF_10_ = 0.34). Finally, self-report problems of EFs on several clinical scales of the BRIEF-A correlated with PM target accuracy in the ASD group but not the NT group. Additionally, there was a trend that PM target accuracy in the ASD group correlated with self-rated PM as well as self-rated retrospective memory.

## Discussion

This study investigated whether emphasising the importance of PM affected task performance in ASD and how changes in performance related to behavioural and self-report measures of EFs, memory, and mentalizing. There were several key findings. Emphasising the importance of the TBPM task over the ongoing task increased PM performance of participants with ASD significantly. In the standard (PM low importance) condition, the ASD group showed diminished PM performance, in keeping with findings from multiple previous studies (Altgassen et al. 2009, 2012; Williams et al. 2013, 2014). Crucially, the change in PM performance among participants with ASD reduced the size of the between-group difference in PM hits from a large (in the PM low importance condition) to a small-to-medium sized effect (in the PM high-importance condition). In comparison, NT participants did not significantly increase their performance from the PM low to the PM high importance condition. Thus, the ASD group seemingly benefitted more from the experimental manipulation than the NT group. However, NT participants performed near ceiling in the PM high importance condition, which makes the true difference in the size of the between-group difference difficult to establish with certainty. What is more important though is how performance in the ASD group in the *PM high importance condition* compares to performance in the NT group in the *PM low importance condition*. This comparison indicates whether individuals with ASD are capable of achieving NT performance levels when using a specific strategy. Indeed, with respect to PM hits, performance among ASD participants in the *high* importance condition was equivalent to performance among NT participants during the *low* importance condition.

To our knowledge, only two previous studies have explored ways to improve TBPM in ASD. Altgassen et al. (2014) explored whether the use of implementation intentions as an explicit memory encoding strategy might improve TBPM in adults with ASD. In a more recent study, Altgassen et al. (2019) explored whether emphasising the importance for someone else (social importance) or receiving a reward (personal importance) would positively affect TBPM in adolescents with ASD. However, neither study found beneficial effects of their experimental manipulation on TBPM performance in ASD compared to NT controls. As such, this study is the first of its kind to show that TBPM in ASD can be improved.

In line with previous studies manipulating relative importance in typical samples (Kliegel et al. 2001; Loft et al. 2008; Smith and Bayen 2004; Walter and Meier 2014), PM improvement in the ASD group was associated with a change in strategic time monitoring, and thus a change in attentional allocation. Specifically, the ASD group increased their frequency of time checks in the PM high importance condition compared to the PM low importance condition. Yet, the increase in the critical time interval prior to target time was lower in the ASD compared to the NT group. Strikingly, the improvement in PM performance did not come at the cost of diminished ongoing task performance among participants with ASD. Thus, it was not the mere reallocation of attention from ongoing task to the PM task that resulted in increased PM performance. Rather, emphasising the importance of the PM task made participants with ASD more likely to remember to carry out the planned action at the appropriate time all the while they were successfully completing the ongoing task.

These results imply that impairments on standard laboratory based TBPM tasks, as well as difficulties with everyday TBPM, among people with ASD arise partly from difficulties with appropriately allocating attention to the PM task at hand. This is likely to reflect, in part, common difficulties with executive control and planning in ASD (Hill 2004; Kenworthy et al. 2008; Lai et al. 2016; Landry and Al-Taie 2016). Indeed, the results from correlation analyses support this interpretation. Among participants with ASD, the extent to which PM hits increased from the PM low to the PM high importance condition was associated significantly with the number of perseverative errors made on the WCST. Hence, the more difficulties the participants had with cognitive flexibility and shifting mindset, the more they benefitted from the emphasised instructions in the PM high importance condition. This is in line with a study that found a correlation between TBPM and switching in children with ASD (Henry et al. 2014) but opposite to Williams et al. (2013) who found no association between WCST performance and TBPM.

Similarly, the extent to which PM accuracy increased from the PM low to the PM high importance condition was associated significantly with self-reported inhibitory control among participants with ASD. Hence, the more problems participants reported with inhibiting prepotent responses, the more they benefitted from the emphasised instructions in the PM high importance condition. This finding was not reflected in a similar correlation with the Stroop inhibition index. It may be that the self-report measure is more sensitive to inhibitory control problems relevant for PM in ASD that do not become evident in a structured lab-based inhibition task. Moreover, self-report inhibitory control problems are associated with independent adaptive functioning and quality of life (de Vries and Geurts 2015; Pugliese et al. 2016) which PM is important for (Henry et al. 2014). Thus, elevated scores on the BRIEF-Inhibit scale above the clinically interesting cut-off might be a tool to identify individuals with ASD who need more support with TBPM.

It is important to note that a large number of correlations were conducted in the current study, which inflates the risk of making type I errors. However, both of these significant correlations would remain significant if Bonferroni corrections for multiple comparisons were applied (although see Perneger 1998, for arguments against applying such corrections) and Bayesian analyses for the significant correlations suggested strongly that the data supported the alternative hypothesis. Overall, then, the results imply that supporting executive control processes by giving explicit instructions can positively support TBPM among people with ASD. These results extend existing research into importance instructions effects and are in line with results from other studies that reported beneficial effects on task performance of explicit instructions among people with ASD (Bowler et al. 2000). More generally, they are in keeping with the “task support hypothesis” (Bowler et al. 1997), which suggests that individuals with ASD experience less memory difficulties when provided with cues to the to be remembered test stimuli. In the present study, a change in strategic time monitoring in the ASD group provided participants with more cues to PM retrieval. Therefore, it may be that the explicit importance instruction provides useful structure and scaffolding in managing dual task demands for participants with ASD leading to improved performance. This could be particularly useful in employment settings to alleviate communication and interactional difficulties (Hendricks 2010), when individuals with ASD are required to manage multi-tasking demands (e.g., finishing a work report and being on time for a work meeting).

The results are also partly consistent with the Triple I hypothesis (White 2013) in that this theory maintains that diminished cognitive task performance in ASD often results from a failure to infer the experimenter’s expectation about what the participant is required to do to succeed on the task. However, contrary to the prediction that would stem from this theory, the extent to which PM performance increased across conditions was not associated significantly with mentalizing ability. If the Triple I hypothesis could explain the TBPM difficulties seen in ASD, then those with the poorest mentalizing abilities should have benefitted the most from the introduction of explicit instructions in the PM high importance condition. The lack of a significant correlation between mentalizing ability in this sample of participants with ASD is not, therefore, consistent with this idea (and, indeed, is contrary to the finding of Williams et al. 2013).

Altogether, the results of this study provide initial evidence that the explicit instruction of the importance of a delayed intention scaffolds TBPM task performance in adults with ASD due to a shift in executive and strategic monitoring processes. Compared to previous attempts to improve TBPM in ASD (Altgassen et al. 2019; Kretschmer et al. 2014), the manipulation of relative importance instructions in the current study resulted in a clear enhancement of PM task performance among participants with ASD. Thus, this approach is so far the most promising strategy to improve TBPM in this disorder. Future research could extend this finding in several ways. The current study found beneficial effects of explicit importance instruction under controlled laboratory conditions. However, a previous study (Altgassen et al. 2012) found significant ASD-specific PM impairments during a naturalistic breakfast preparation task. It would be interesting to establish whether the effect of importance instructions applies equally under real-life/naturalistic task demands as under laboratory task demands among people with ASD. Furthermore, previous research has found that PM decreases with age, which can be alleviated (e.g.; Hering et al. 2014; Zimmermann and Meier 2010). Thus, it would be important to compare younger and older adults with ASD to explore whether the importance manipulation is equally effective across different ages. Finally, it would be important to explore whether the execution of self-generated compared to other-generated (family, work colleague, experimenter) intentions differs in ASD. Related to this, future research should also explore how self-rated (personal) importance of intentions (Ihle et al. 2012; Niedźwieńska et al. 2013) affects PM performance. Exploration of these factors would provide further important insight into how to tailor task instructions in order to maximally benefit task performance of individuals with ASD.

## Electronic supplementary material

Below is the link to the electronic supplementary material.

Supplementary material 1 (DOCX 57 kb)

## Footnotes

1 It is not possible within JASP to calculate the BF_10_ for the model which is indicated based on traditional null hypothesis significance testing: main effects of condition and interval, two-way interactions between Group × Interval, and Importance × Interval, and the three-way interaction Group × Importance × Interval of Importance. Therefore, it is difficult to estimate what the additional three-way interaction adds to the model in comparison to a model without it.
