# Supplementary material for: Increasing Extrinsic Motivation Improves Time-Based Prospective Memory in Adults with Autism: Relations with Executive Functioning and Mentalizing
Source: J Autism Dev Disord. 2019 Dec 21;50(4):1133–46. doi: 10.1007/s10803-019-04340-2 (PMC7101298; doi:10.1007/s10803-019-04340-2)
Supplement: Supplementary file 1 — Supplementary material 1 (DOCX 57 kb) [file 10803_2019_4340_MOESM1_ESM.docx]

# Supplementary Materials

S1) Relative Importance Effects and Event-based Prospective Memory

More information on the relative importance manipulation in studies of event-based PM can be found below:

In a second experiment, Kliegel et al. (2001) manipulated relative importance in an event-based PM task. They explored whether task importance had an effect when PM performance did not require a high degree of attentional resources. For this purpose, they used highly salient and focal PM cues, which promoted PM cue detection. The ongoing task was the same word rating task that they used for their TBPM experiment. They found that importance did neither affect PM task nor ongoing task performance. This suggests that when PM is mediated by automatic processes, no additional strategic attentional allocation is necessary to perform well.

Other studies have investigated importance effects on event-based PM under more demanding task conditions (Loft, Kearney, & Remington, 2008; Smith & Bayen, 2004). Smith and Bayen (2004) examined the effects of relative importance under high retrospective memory load. Participants had to remember six PM target words to respond to during a colour-matching task. Participants saw coloured rectangles presented sequentially prior to the appearance of a word. The task was to indicate whether the word colour matched any of the previously seen rectangles’ colours. They found that high PM importance increased PM accuracy. This resulted in slowed reaction times in the OT, however. Loft et al. (2008) studied the effects of relative importance on performance depending on the actual occurrence of PM targets. Besides the established finding that importance increased PM hit rates, they found that monitoring costs (RT slowing) were reduced in task blocks with no PM targets compared to task blocks with PM targets.

Altogether, the above findings indicate that stressing the importance of a PM task increases performance due to a strategic shift in attention allocation processes. This, however, can cause interference with OT performance. Importantly, in line with the multiprocess framework (McDaniel & Einstein, 2000), the effects of importance instructions will vary depending on OT demands and characteristics of the PM cue. If the PM task success heavily relies on attentional resources, importance instructions will benefit performance. If PM is mediated by automatic processes, importance instructions should not make a difference (Kliegel et al. 2004).

S2) Details on Cognitive-Behavioural Tasks

*Stroop task details:* Participants initially completed a short practice to familiarise themselves with the response options (which keyboard key corresponded to which word colour). Subsequently, participants completed 150 trials (50 per condition). Word stimuli in the congruent and incongruent condition entailed four colour words (BLUE, GREEN, RED, YELLOW) as well as four neutral non-colour words (CHIEF, MEET, PLENTY, TAX). During data analysis, trials with reaction times faster than 200ms (anticipatory) or slower than 2.5SDs above the individual mean reaction time across all trials were excluded from the analysis.

*Wisconsin Card Sorting Task details:* The task materials consisted of four stimulus cards and 128 response cards which varied on three dimensions: number of shapes (1 to 4), type of shape (circle, cross, star, triangle), and colour of shapes (blue, green, red, yellow) with each card showing a different, shape, colour, and number of shape. The participant’s task was to sort each response card presented below the stimulus cards into categories. Participants had to determine what the sorting rule was (sorting by type of shape, number of shape or colour) and received feedback whether they responded correctly or not after each trial. The card that needed to be categorized never matched on more than one feature with each of the four stimulus cards (e.g., shape and colour). The sorting rule changed unbeknown to the participant after 10 cards were sorted correctly. The number of perseverative errors was used as an index of cognitive inflexibility (i.e., a tendency to become stuck in set). A perseverative error was defined as persisting to sort a card into the same category as the previous one, after they had received feedback of their previous sort being incorrect (see Cianchetti et al. 2007) . This measure has been found to reliably distinguish ASD from NT individuals (Landry & Al-Taie, 2016).

The Stroop test and WCST were chosen as established standard behavioural tests of EFs in neurodevelopmental disorders (Ozonoff & Jensen, 1999). Both the WCST and Stroop tests have been linked to performance on TBPM tasks in NT individuals and/or individuals with ASD (Henry et al. 2014; Kliegel et al. 2002; Kliegel et al. 2003; Mioni & Stablum, 2014; Vanneste et al. 2016; Williams et al. 2013). Therefore, these tasks were thought to be suitable to investigate whether performance changes in TBPM from the PM low importance to the PM high importance condition were associated with EF abilities.

*Animations task details:* This task involved watching four short video clips in pseudorandom order. Each clip showed the interaction between a small blue and a big red triangle moving around the screen. Participants had to describe what was happening in each clip. An accurate description of each scene required the attribution of mental states to the triangles (e.g., coaxing or surprising). Participants watched each clip twice and gave a running commentary during the second viewing. Descriptions were recorded, and later transcribed and coded by two independent raters who were unaware of the hypotheses of the study or to group membership. Transcripts were awarded a score of two, one, or zero based on how accurately the descriptions reflected each clip following the coding guidelines provided by Abell, Happé, and Frith (2000). Hence, mentalizing sum scores ranged from zero to eight. Inter-rater reliability was good according to established criteria (Fleiss, Levin, & Paik, 2003; Landis & Koch, 1977), weighted κ = 0.63, *p* < .001.

S3) Descriptives of the BRIEF-A

*Table S1. Sample characteristics for all scales of the BRIEF-A*

|  | **Group means (SD)** | |  |  |  |  |
| --- | --- | --- | --- | --- | --- | --- |
|  | **ASD (*n* = 25)** | **NT (*n* = 22)** | ***T*** | ***df*** | ***p*** | **Cohen's *d*** |
| **Clinical scales** |  |  |  |  |  |  |
| *BRIEF Inhibit* | 63.40 (12.47) | 51.82 (9.81) | -3.50 | 45 | .001 | 1.17 |
| *BRIEF Shift* | 72.04 (13.82) | 50.59 (8.16) | -6.37 | 45 | <.001 | 2.07 |
| *BRIEF Emotional Control* | 61.80 (16.70) | 47.64 (6.47) | -3.74 | 45 | .001 | 1.01 |
| *BRIEF Self-monitoring* | 62.72 (14.36) | 46.18 (10.01) | -4.52 | 45 | <.001 | 1.51 |
| *BRIEF Initiate* | 65.72 (13.93) | 51.27 (10.42) | -3.98 | 45 | <.001 | 1.48 |
| *BRIEF Working memory* | 72.96 (12.97) | 52.82 (10.97) | -5.71 | 45 | <.001 | 1.83 |
| *BRIEF Plan* | 65.24 (13.46) | 52.41 (12.48) | -3.37 | 45 | .002 | 1.23 |
| *BRIEF Task monitoring* | 65.88 (14.39) | 53.00 (11.46) | -3.36 | 45 | .002 | 1.20 |
| *BRIEF Organisation of materials* | 56.44 (14.45) | 46.73 (8.99) | -2.72 | 45 | .009 | 0.85 |
| **Composite scores** |  |  |  |  |  |  |
| *BRIEF Behavioural Regulation Index* | 68.24 (13.88) | 48.64 (6.73) | -6.03 | 45 | <.001 | 2.04 |
| *BRIEF Metacognition Index* | 68.00 (12.94) | 51.68 (11.56) | -4.53 | 45 | <.001 | 1.60 |
| *BRIEF Global Executive Composite* | 69.52 (13.51) | 51.82 (9.81) | -5.63 | 45 | <.001 | 2.01 |

S4) Subsample Analysis of Groups Matched for Ongoing Performance

Due to the dual-task nature of PM tasks, attentional resources are divided between ongoing and PM task. As a result, between-group differences in OT performance might reflect that fewer resources were available to perform the PM task. Thus, to interpret PM performance in the ASD group, it is important that their OT performance is matched with the NT group. Therefore, prior to the analysis of PM task performance, the group difference in proportion of hits on the OT was analysed. For this purpose, OT performance was collapsed across conditions and entered into an independent-samples t-test with group as the independent variable. This analysis revealed that the ASD group (M = 0.64, SD =0.13) performed significantly worse than the control group (M = 0.76, SD = 0.11), t(46) = 3.55, p = .001, d = 0.99. Since these group differences could have influenced PM performance in the different conditions, all analyses were repeated among a subsample that was matched for OT performance as well as baseline characteristics. In order to achieve this matching, the NT participants with the highest OT performance (*n =4)* and the ASD participants (*n = 6)* with the lowest OT performance were excluded sequentially. This process continued until no group differences emerged and the corresponding effect size was small, *t*(36) = 1.40, *p* = .17, *d* = - 0.45. This resulted in sub-groups of 19 participants (3 females in each group respectively) who were matched for age and IQ (all *p*s ≤ .05, all *d*s ≤ 0.53, see Table A2). Crucially, the results concerning PM performance were not substantially different in this subsample (matched for OT performance) and in the full sample (unmatched for OT performance. Therefore, the results from the full sample are reported here.

*Table S2. Group characteristics of sub-sample matched with groups matched for ongoing task performance (collapsed across conditions)*

|  | **Group means (SD)** | |  |  |  |  |
| --- | --- | --- | --- | --- | --- | --- |
|  | **ASD**  **(*n* = 19, 1 female)** | **NT**  **(*n* =19, 3 female)** | ***T*** | ***df*** | ***p*** | **Cohen's *d*** |
| **Age** | 34.26 (12.60) | 39.15 (12.52) | 0.953 | 36 | .347 | -0.31 |
| **VIQ** | 107.68 (13.00) | 102.00 (7.98) | -1.624 | 36 | .113 | 0.53 |
| **PIQ** | 107.63 (18.71) | 104.21 (11.19) | -0.684 | 36 | .498 | 0.22 |
| **FSIQ** | 108.21 (15.59) | 103.37 (8.91) | -1.176 | 36 | .247 | 0.38 |
| **AQ** | 31.53 (9.23) | 17.74 (6.90) | -5.214 | 36 | <.001 | 1.69 |
| **ADOS** | 7.95 (3.55) | - | - | - | - | - |

Ongoing task

Ongoing proportion hit scores were entered into a 2 × 2 mixed ANOVA with group (ASD/NT) as between-subject factor and condition (PM low/high importance) as within-subject factor. The main effect of condition was significant, *F*(1,36) = 6.62, *p* = .01, $\eta_{p}^{2}$ = .16, BF_10_ = 3.42 with better ongoing performance in the *PM low importance condition*. No significant main effect of group or interaction effect emerged (all *F*s ≤ 1.96, all *p*s ≥ .17, all $\eta_{p}^{2}$ ≤ .05, all BF_10_ ≤ 0.77; see Table A3). Neither, the full (BF_10_ = 0.87) nor the additive (BF_10_ = 2.69) model of the Bayesian analysis fitted the data better than the main effect of condition only.

PM task

The Group (ASD/NT) × Condition (PM low/high importance) mixed ANOVA on PM proportion hit scores revealed a main effect of group, *F*(1,36) = 7.35, *p* = .01, $\eta_{p}^{2}$ = .17, BF_10_ =4.64, with the ASD group performing worse overall than the NT group, and a main effect of condition, *F*(1,36) = 12.83, *p* = .001, $\eta_{p}^{2}$ = .26, BF_10_ = 20.25, indicating better PM performance in the PM high importance condition. Importantly, these effects were qualified by a significant group by condition interaction, *F*(1,36) = 5.39, *p* = .03, $\eta_{p}^{2}$ = .13, BF_10_ = 2.31. The BF_10_ for the additive model was 101.04 and for the full model 232.34. This means that the full model provided the greatest evidence for the present data. Post-hoc tests revealed that only the ASD group showed a significant increase in PM hit proportion scores from the PM low to the PM high importance condition (ASD: *F*(1,36) = 17.43, *p* < .001, $\eta_{p}^{2}$ = .33, BF_10_ = 8.92; NT: *F*(1,36) = .79, *p* =.38, $\eta_{p}^{2}$ = .02, BF_10_ = 0.83). Tests of simple effects of indicated that the NT group outperformed the ASD group in the PM low importance condition, *F*(1,36) = 9.49, *p* = .004, $\eta_{p}^{2}$ = .21, BF_10_ = 10.30. In the PM high importance condition, however, PM performance did not differ significantly between groups, *F*(1,36) = 1.92, *p* = .17, $\eta_{p}^{2}$ = .05, BF_10_ = 0.67 (see Ongoing and PM performance scores by condition of sub-sample matched with groups matched for ongoing task performance (collapsed across conditions)Table A3 for means and SD). However, both groups performed at ceiling in the PM high importance condition, ASD: *t*(18) = =1.61, *p* = .13, NT: *t*(18) = -1.00, *p* = .33; thus cautious interpretation of the interaction effect is required.

With regard to PM target accuracy a Group (ASD/NT) × Condition (PM low/high importance) mixed ANOVA was performed on the data of those participants who at a minimum of one PM hit (low importance both groups *n* = 23, high importance ASD: *n* = 18, TD: *n* = 19). A main effect of group, *F*(1,35) = 5.28, *p* = .03, $\eta_{p}^{2}$ = .13, BF_10_ = 2.34, emerged indicating that NT group overall responded closer to target time than the ASD group (see Table A3). Neither the main of condition nor the interaction were significant (all *F*s ≤ 2.76, all *p*s ≥ .11, all $\eta_{p}^{2}$ ≤ .07, all BF_10_ ≤ 0.74). The BF_10_ for the additive model was 1.73 and for the full model 0.81.

*Table S3.* *Ongoing and PM performance scores by condition of sub-sample matched with groups matched for ongoing task performance (collapsed across conditions)*

|  |  | **Group means (SD)** | |  |
| --- | --- | --- | --- | --- |
| **Condition** |  | **ASD** | **NT** | **Cohen’s *d*** |
| *PM low importance* | Ongoing proportion hits | .71 (0.11) | .75 (0.09) | -0.40 |
|  | PM proportion hits | .72 (0.32) | .95 (0.09) | -0.98 |
|  | PM target accuracy | 2.20 (1.67) | 1.26 (0.89) | 0.70 |
| *PM high importance* | Ongoing proportion hits | .67 (0.11) | .71 (0.12) | -0.35 |
|  | PM proportion hits | .91 (0.24) | .99 (0.05) | -0.46 |
|  | PM target accuracy | 1.66 (0.98) | 1.13 (0.89) | 0.57 |

Time-monitoring

A 2 ×2 × 4 mixed ANOVA was conducted on the mean number of time checks carried out during the task with group (ASD/NT) as between-subject factor, and condition (PM low/high importance) and time interval (0-30, 31-60, 61-90, 91-120 seconds) as within-subject factors. Mauchly’s test indicated that the assumption of sphericity had been violated for the main effect of interval and the interval × importance interaction effect. Therefore, Greenhouse-Geisser correction was used for those effects. No main effect of group was found, *F*(1,36) = 0.75, *p* = .39, $\eta_{p}^{2}$ = .02, BF_10_ = 0.28. There were significant main effects of condition, *F*(1,36) = 15.89, *p* < .001, $\eta_{p}^{2}$ = .31, BF_10_ = 20.22, and interval *F*(1.35,48.58) = 94.55, *p* < .001, $\eta_{p}^{2}$ = .72, BF_10_ = 1.25*10^40^. The overarching three-way interaction Group × Importance × Interval was not significant, *F*(2.04,73,32) = 1.42, *p* = .25, $\eta_{p}^{2}$ = .04, BF_10_ = 0.15, however some two-way interactions were. The significant interaction between group and interval *F*(1.35,48.58) = 5.58, p = .01, $\eta_{p}^{2}$ = .13, BF_10_ = 124.07, indicated that the NT group checked the time more often than the ASD group. However, tests of simple effects only revealed a marginally significant group difference for the interval prior to target time (91-120 seconds) with more time checks in the NT group (*M*=2.75, *SE* =.27) in comparison to the ASD group (*M* = 2.02, *SE* = .27), *F*(1,36) = 3.53, *p* = .06, $\eta_{p}^{2}$ = .09, BF_10_ = 1.29. Furthermore the Condition × Interval interaction, *F*(2.04,73.32) = 6.51, *p* = .002, $\eta_{p}^{2}$ = .15, BF_10_ = 1.77, revealed that for three intervals (31-60, 61-90, 91-120 seconds) across both groups, participants checked the time significantly more often in the high importance condition than in the low importance condition (all *p*s ≤ .001, all $\eta_{p}^{2}$ ≥ .50, all BF_10_ ≥ 3.65).. The Group × Importance interaction was not significant, *F*(1,36) = 0.10, *p* = .76, $\eta_{p}^{2}$ = .003, BF_10_ = 0.18.

References

Abell, F., Happé, F., & Frith, U. (2000). Do triangles play tricks? Attribution of mental states to animated shapes in normal and abnormal development. *Cognitive Development, 15*(1), 1-16. doi:10.1016/s0885-2014(00)00014-9

Cianchetti, C., Corona, S., Foscoliano, M., Contu, D., & Sannio-Fancello, G. (2007). Modified Wisconsin Card Sorting Test (MCST, MWCST): normative data in children 4-13 years old, according to classical and new types of scoring. *The Clinical neuropsychologist, 21*(3), 456-478. doi:10.1080/13854040600629766

Fleiss, J. L., Levin, B., & Paik, M. C. (2003). *Statistical Methods for Rates and Proportions* (3rd ed.). Hoboken, New Jersey: Wiley.

Kliegel, M., Martin, M., McDaniel, M. A., & Einstein, G. O. (2001). Varying the importance of a prospective memory task: Differential effects across time- and event-based prospective memory. *Memory, 9*(1), 1-11. doi:10.1080/09658210042000003

Kliegel, M., Martin, M., McDaniel, M. A., & Einstein, G. O. (2002). Complex prospective memory and executive control of working memory: A process model. *Psychologische Beitraege, 44*, 303-318.

Kliegel, M., Martin, M., McDaniel, M. A., & Einstein, G. O. (2004). Importance effects on performance in event-based prospective memory tasks. *Memory, 12*(5), 553-561. doi:10.1080/09658210344000099

Kliegel, M., Ramuschkat, G., & Martin, M. (2003). Exekutive Funktionen und prospektive Gedächtnisleistung im Alter - Eine differentielle Analyse von ereignis- und zeitbasierter prospektiver Gedächtnisleistung. *Zeitschrift fur Gerontologie und Geriatrie, 36*(1), 35-41. doi:10.1007/s00391-003-0081-5

Landis, J. R., & Koch, G. G. (1977). The measurement of observer agreement for categorical data. *Biometrics, 33*(1), 159-174. doi:10.2307/2529310

Landry, O., & Al-Taie, S. (2016). A Meta-analysis of the Wisconsin Card Sort Task in Autism. *J Autism Dev Disord, 46*(4), 1220-1235. doi:10.1007/s10803-015-2659-3

Loft, S., Kearney, R., & Remington, R. (2008). Is task interference in event-based prospective memory dependent on cue presentation? *Memory & Cognition, 36*(1), 139-148. doi:10.3758/Mc.36.1.139

McDaniel, M. A., & Einstein, G. O. (2000). Strategic and automatic processes in prospective memory retrieval: A multiprocess framework. *Applied Cognitive Psychology, 14*(7), S127-S144. doi:10.1002/acp.775

Mioni, G., & Stablum, F. (2014). Monitoring behaviour in a time-based prospective memory task: The involvement of executive functions and time perception. *Memory, 22*(5), 536-552. doi:10.1080/09658211.2013.801987

Ozonoff, S., & Jensen, J. (1999). Brief report: Specific executive function profiles in three neurodevelopmental disorders. *Journal of Autism and Developmental Disorders, 29*(2), 171-177. doi:10.1023/a:1023052913110

Smith, R. E., & Bayen, U. J. (2004). A multinomial model of event-based prospective memory. *Journal of Experimental Psychology: Learning, memory, and cognition, 30*(4), 756-777. doi:10.1037/0278-7393.30.4.756

Vanneste, S., Baudouin, A., Bouazzaoui, B., & Taconnat, L. (2016). Age-related differences in time-based prospective memory: The role of time estimation in the clock monitoring strategy. *Memory, 24*(6), 812-825. doi:10.1080/09658211.2015.1054837

Williams, D. M., Boucher, J., Lind, S. E., & Jarrold, C. (2013). Time-based and event-based prospective memory in autism spectrum disorder: The roles of executive function and theory of mind, and time-estimation. *Journal of Autism and Developmental Disorders, 43*(7), 1555-1567. doi:10.1007/s10803-012-1703-9.
